# Phytochemical Characterization and In Vitro and In Silico Biological Studies from Ferns of Genus *Blechnum* (Blechnaceae, Polypodiales)

**DOI:** 10.3390/antiox12030540

**Published:** 2023-02-21

**Authors:** Alfredo Torres-Benítez, José Erick Ortega-Valencia, Mathias Flores-González, Marta Sánchez, Mario J. Simirgiotis, María Pilar Gómez-Serranillos

**Affiliations:** 1 Instituto de Farmacia, Facultad de Ciencias, Universidad Austral de Chile, Campus Isla Teja, Valdivia 5090000, Chile; 2 Tecnológico Nacional de México, Instituto Tecnológico de Tlalnepantla, Av. Instituto Tecnológico, S/N. Col. La Comunidad, Tlalnepantla de Baz 54070, Mexico; 3 Departamento de Farmacología, Farmacognosia y Botánica, Facultad de Farmacia, Universidad Complutense de Madrid, Plaza Ramón y Cajal s/n, Ciudad Universitaria, 28040 Madrid, Spain

**Keywords:** *Blechnum*, bioactive compounds, antioxidant, enzyme inhibition, pharmacological potential, ferns

## Abstract

The genus *Blechnum* represents one of the most ecologically and therapeutically important groups of ferns that grow in tropical, subtropical and temperate regions. In this work, the chemical fingerprint of lyophilized extracts of *Blechnum chilense, B. hastatum, B. magellanicum* and *B. penna-marina* species, the determination of their antioxidant activity through ORAC, FRAP and DPPH assays and inhibition of cholinesterase enzymes (AChE and BChE), and an in silico analysis of selected majority compounds on cholinesterase enzymes were identified. Nineteen compounds were recorded for *B. chilense*, nine in *B. hastatum,* seventeen in *B. magellanicum* and seventeen in *B. penna-marina* by liquid chromatography coupled with quadrupole-time-of-flight mass spectrometry (UHPLC-ESI-QTOF-MS). The content of phenolic compounds, flavonoids, antioxidant activity and enzyme inhibition were variable among species, with best results for *B. penna-marina*. Molecular docking evidenced low toxicities, significant pharmacokinetic properties, and significant binding affinities of the tested compounds for the AChE and BChE enzymes. These fern species show high diversity of bioactive compounds and represent a promising resource in phytotherapy, especially for their optimal levels of phenolic compounds that support their antioxidant activity.

## 1. Introduction

Oxidative stress can be defined as a complex biological process in which there is an imbalance between the amount of free radicals present in the body and the capacity to eliminate them, which are the product of metabolic processes involving oxygen, initiating the formation of reactive oxygen species (ROS). Phenolic compounds such as flavonoids are widely distributed in plants with a high antioxidant capacity due to the action of hydroxyl groups present in their chemical structure that neutralize ROS [1,2].

Products derived from medicinal plants, due to their therapeutic qualities, have become a raw material not only in research, but also an important resource in the pharmaceutical industry. Ferns and related plants are a widely distributed plant group and several species are used in traditional medicine for respiratory and intestinal diseases, among others [3,4,5]. Studies in recent years report in this group a diversity of compounds such as alkaloids, diterpenes, triterpenes, flavonoids, polyphenols and steroids that represent a potential bioactivity, especially in species of the families Huperziaceae, Blechnaceae, Pteridaceae, Dryopteridaceae, Thelypteridaceae, Polypodiaceae, Aspleniaceae, Gleicheniaceae, Dicksoniaceae, Davalliaceae and others [6,7,8]. Ferns of the genus *Blechnum* L. are widely distributed worldwide and are a promising source of bioactive compounds (phenolic acids, lignans, flavonols, flavones, anthocyanidins, sesquiterpene, diterpenes, sterols, fatty acids, alcohols, aldehydes, carotenoids and heterocyclic) with antipyretic, analgesic, anti-inflammatory, antimicrobial and antioxidant properties [9,10,11].

The objective of this work was to identify the bioactive compounds of freeze-dried extracts of the ferns *B. chilense, B. hastatum, B. magellanicum* and *B. penna-marina*, and determine their antioxidant activity and inhibition of cholinesterase enzymes through in vitro assays and molecular docking of compounds present in the extracts of the species.

### 1.1. Botanical and Taxonomic Description

The genus *Blechnum* L. is in the family Blechnaceae (order Polypodiales) with approximately 236 species accepted worldwide that are considered cosmopolitan and chromosomal compositions of 2n = 28, 29, 31, 32, 33, 34, 36 that have been reported (http://www.worldfloraonline.org, accessed on 25 January 2023).

**Figure 1:**
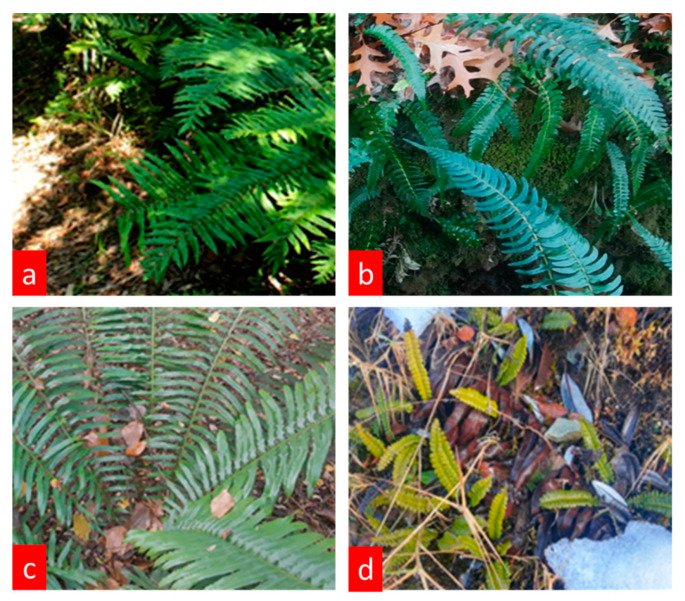
(**a**) B. chilense; (**b**) B. hastatum; (**c**) B. magellanicum; (**d**) B. penna-marina.

The specie *B. chilense* (Kaulf.) Mett. is characterized by leaves from 15 to 40 cm long, petiole of 1 mm in diameter, brown, shiny, half of half diameter, brown with black, shiny, half of the total length of the total leaf length; lamina deltoid, bipinnate to tripinnate. The last segments are glabrous, petiolate, coriaceous, subtrapezoidal or reniform, at the base sometimes cuneate, the upper edge divided into 4 to 7 broad, shallow, slightly denticulate, broadly slightly denticulate; veins numerous, divided 1 or 2 times, flabellate. Sori marginal, reniform or somewhat elongated, 1 to 3 mm long, protected by the 3 mm long, protected by the indusium originating from the segment margin, which has by the margin of the segment, which has a hemispherical central hemispherical notch [12] (Figure 1a).

The specie *B. hastatum* Kaulf. is characterized by pinnate, monomorphous leaves, usually 10 to 70 cm long; petiole 3 to 25 cm long, with 3 to 25 cm long, with scales at the base, chaedaceous; lamina subherbaceous to coriaceous, oval-lanceolate in outline, elongated and acute apex, broad base truncate to blunt; rachis with scattered multicellular hairs; pinnae glabrous or with sparse scattered hairs, more or less auriculate at the base, attached to the rachis by a short petiolule; apical pinnae gradually adnate to the rachis. Sori in cenospores submarginal, occupying three-fourths or more of the length of the pinna, often interrupted; indusium papiraceous, lateral, whitish, somewhat lacerate [12] (Figure 1b).

The specie *B. magellanicum* (Desv.) Mett. is characterized by a densely scaly, woody rhizome with pinnate, dimorphic leaves; the sterile ones are 0.5 to 1.5 m long; the petiole is subleaf, from 15 to 35 cm long; petiole subwoody, densely covered at the base with dark brown scales, resembling hairs; lamina, oblong to oblong-lanceolate, coriaceous; pinnae alternate or subopposite, 8 to 15 cm long by 0.8 to 1.5 cm wide, acute, adnate to the rachis by a wide base base; erect fertile leaves, equal or sometimes shorter than the sterile ones; narrow pinnae about 5 mm wide. Sori in cenospores that entirely cover the underside of the pinna when mature; lateral indusium, continuous [12] (Figure 1c).

The specie *B. penna-marina* (Poiret) Kuhhn characterized by erect rhizome or sometimes somewhat creeping, pinnate leaves, dimorphic, petioles agglomerated, dark brown to almost black, of the less than half the total leaf length, with some scales at the base; sterile, 2 to 25 cm long, linear-lanceolate; rachis with lanceolate scales, light brown, scattered, chaedulate; pinnae subopposite, glabrous, subcoriaceous to membranous, 3 to 5 cm long and by 2 to 5 mm wide, oblong to triangular, obtuse to subacute, arranged perpendicular to the rachis; fertile leaves up to 3 times longer than sterile ones; pinnae linear than the sterile ones; pinnae linear to oblong, more or less curved, well-spaced, sometimes with a basal lobe. Sori in continuous cenospores, covering almost the entire lower surface of pinna, indusium continuous [12] (Figure 1d).

### 1.2. Distribution

**Figure 2:**
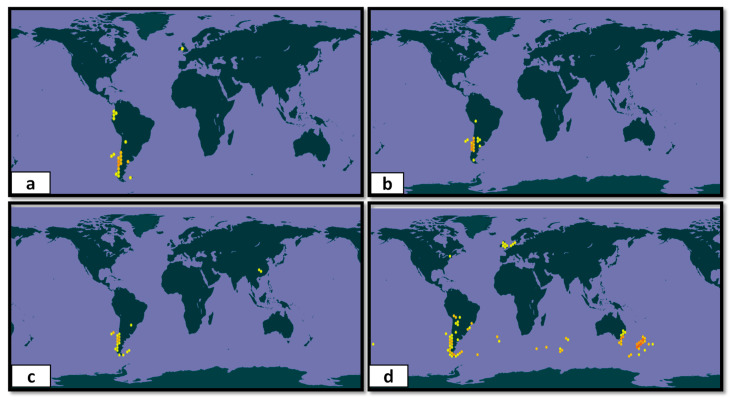
Distribution of Blechnum species (GBIF): (**a**) B. chilense; (**b**) B. hastatum; (**c**) B. magellanicum; (**d**) B. penna-marina.

The species of the genus *Blechnum* are distributed in the five continents and oceanic islands (https://powo.science.kew.org/, accessed on 25 January 2023). The species *B. chilense* is native to Chile and Argentina, and it also grows in the Juan Fernandez archipelago. They are ferns of very humid sites and can be found associated with species of the genus *Gunnera* and usually invade the interior of forests, especially in the ravines. The species *B. hastatum* is native to Chile and Argentina and is also found in the archipelago of Juan Fernandez, grows in open places, under shrubs and near streams. The species *B. magellanicum* is native to Chile and Argentina, and is a fern inside the forest, in shady and humid places such as ravines. The species *B. penna-marina*, which has a circumpolar southern distribution with preferences in island areas and is found in Argentina, Brazil, Bolivia, Chile, Australia and islands of the Pacific, Indian and South Atlantic oceans, is a fern associated with moist places in the undergrowth [12] (Figure 2).

## 2. Materials and Methods

### 2.1. Chemicals 

HPLC-grade methanol and MS-grade formic acid were used for mass spectrometric analysis (J.T. Baker, Phillipsburg, NJ, USA). Ultrapure water (˂5 µg/L TOC) was obtained from a water purification system (Mili-Q Merck Millipore, Chile). Gallic acid, sodium carbonate, Commercial Folin Ciocalteu reagent, sodium acetate, acetic acid, 2,4,6-tris(2-pyridyl)-s-triazine, hydrochloric acid, 2,2-diphenyl-1-picrylhydrazyl, Trolox, quercetin, sodium nitrite solution, aluminum chloride, ferric chloride hexahydrate, phosphate buffer, absolute ethanol, 2,2′-Azobis(2-amidinopropane) dihydrochloride, fluorescein solution, acetylcholinesterase (AChE) enzyme, butyrylcholinesterase (BChE) enzyme, acetylcholine, Ellman’s reagent (DTNB), galantamine, butyrylcholine, magnesium chloride, sodium chloride, solution Tris-HCl buffer were obtained from the supplier Sigma-Aldrich (St. Louis, MO, USA).

### 2.2. Ferns Material

The species *B. chilense* and *B. hastatum* were collected in the botanical garden of the Universidad Austral de Chile in the city of Valdivia, Los Ríos region (Chile). The species *B. magellanicum* was collected near Oncol Park, Valdivia province, Los Ríos region (Chile). The species *B. penna-marina* was collected in the locality of Camarones, Osorno province, Los Lagos region (Chile). For taxonomic identification, morphological characters were used based on bibliographic sources, and specimens were determined by botanist Alfredo Torres-Benítez and the support of the Botanical Institute of the Universidad Austral de Chile (Valdivia, Chile).

### 2.3. Preparation of the Lyophylized Extracts

About 10 g of previously ground of each fern species was mixed with 250 mL of distilled water in an Erlenmeyer, then placed in a BIOBASE ultrasonic bath (sonicator) at a frequency of 80 kHz for 30 min at 60 °C in darkness. Subsequently, the solution was filtered with special Fisherbrand™ 602 medium filtering paper and proceeded to lyophilization in a FreeZone LABCONCO benchtop lyophilizer. Finally, the lyophilized material was collected, labeled and stored under refrigeration until use. The final yields for the species were 7% in *B. chilense*, 6% in *B. hastatum*, 4% in *B. magellanicum* and 24% in *B. penna-marina.*

### 2.4. LC Parameters and MS Parameters

With a UHPLC-ESI-QTOF-MS system, equipped with UHPLC Ultimate 3000 RS with Chromeleon 6.8 software (Dionex GmbH, Idstein, Germany), and a Bruker maXis ESI-QTOF-MS with the software Data Analysis 4.0 (all Bruker Daltonik GmbH, Bremen, Germany) we carried out the separation and identification of secondary metabolites from the ferns’ extracts. The chromatographic equipment consisted of a quaternary pump, an autosampler, a thermostated column compartment and a photodiode array detector. We dissolved 5 mg of each extract of fern in 2 mL of methanol for analysis and filtered with a polytetrafluoroethylene (PTFE) filter, and 10 µL was injected into the equipment. Elution was performed with a binary gradient system with eluent (A) 0.1% formic acid in water, eluent (B) 0.1% formic acid in acetonitrile: 1% B isocratic (0–2 min), 1–5% B (2–3 min), 5% B isocratic (3–5 min), 5–10% B (5–8 min), 10–30% B (8–30 min), 30–95% B (30–38 min) and 1% B isocratic (38–50 min). ESI-QTOF-MS experiments were recorded in negative ion mode, and the scan range was between 100 and 12,000 *m*/*z*. Separation was carried out with a Thermo 5 µm C18 80 Å column (150 mm × 4.6 mm) at a flow rate of 1.0 mL/min. Electrospray ionization (ESI) conditions included a capillary temperature of 200 °C, a capillary voltage of 2.0 Kv, a dry gas flow rate of 8 mL/min and a nebulizer pressure of 2 bar, and the experiments were performed in automatic MS/MS mode. The structural characterization of secondary metabolites was based on HR full MS, fragmentation patterns of the ions of compounds and comparisons with bibliography.

### 2.5. Total Phenolic and Total Flavonoid Content

For total phenolic content, the sample of each extract was mixed with distilled water, 10% Folin–Ciocalteu reagent and stored in the dark for 5 min at 37 °C, a 7% Na_2_CO_3_ solution was added and incubated for 30 min in darkness, absorbance at 765 nm was measured in a microplate reader (BioTek Instrument, Inc., Winooski, VT, USA), the results were expressed as mg of gallic acid per gram of dried fern and gallic acid was used as the reference compound [13]. Total flavonoid content was measured using the AlCl_3_ method, absorbance at 510 nm was measured in a microplate reader (BioTek Instrument, Inc., Winooski, VT, USA), the results were expressed as mg of quercetin per gram of dried fern, and quercetin was used as the reference compound [14].

### 2.6. Antioxidant Activity 

#### 2.6.1. Ferric-Reducing Antioxidant Power (FRAP) Assay

The sample of each extract was mixed with a working solution (buffer solutions, FeCl_3_, and TPTZ (2,4,6-tris(2-pyridyl)-s-triazine)), incubated for 5 min, and absorbance was measured at 593 nm in a microplate reader (BioTek Instrument, Inc., Winooski, VT, USA). The results were expressed as micromoles of Trolox equivalents per gram of dried fern. Trolox was used as the reference compound [15].

#### 2.6.2. Oxygen Radical Absorbance Capacity (ORAC) Assay

Fluorescein solution was added to each extracted sample and incubated for 30 min in the dark at 37 °C, then AAPH (2,2’-azobis(2-amidinopropane) dihydrochloride) solution was added, and the excitation and emission wavelengths were measured at 485 and 530 nm, respectively, every 2 min for 1 h and 30 min in a microplate reader (BioTek Instrument, Inc., Winooski, VT, USA). The results were expressed as micromoles of Trolox equivalents per gram of dried fern. Trolox was used as the reference compound [16].

#### 2.6.3. DPPH Scavenging Activity

DPPH solution was added to the solutions of each extract and incubated for 30 min in the dark, and the absorbance was measured at 515 nm in a microplate reader (BioTek Instrument, Inc., Winooski, VT, USA). The results were expressed as IC_50_ values (µg fern/mL). Gallic acid was used as a positive control [17].

### 2.7. Determination of Cholinesterase Inhibition

It was performed according to Ellman’s method. Solutions of the extracts were mixed with Tris-HCl buffer, acetylcholinesterase or butyrylcholinesterase (as appropriate), DTNB solution, incubated for 20 min in the dark at 37 °C, acetylthiocholine or butyryl- thiocholine was added. Absorbance was measured at 412 nm every 2 min for 20 min in a microplate reader (BioTek Instrument, Inc., Winooski, VT, USA). The results were expressed as IC_50_ values (µg fern/mL). Galantamine was used as a positive control [18].

### 2.8. Calculation of ADME Parameters

To determine if the compounds obtained from the *Blechnum* extracts are viable to be used as acetylcholinesterase and butyrylcholinesterase inhibitor candidates, the pharmacokinetic properties were calculated using the Osiris Data Warrior (v 5.5.0) computational tool. The partition coefficient (cLogP), the number of hydrogen bond donors, the number of hydrogen bond acceptors, the molecular mass of the compounds, the topological polar surface area (TPSA) and the number of rotable bonds were calculated to determine the violations of the Lipinski’s rules that the compounds could present and to predict which compounds will have a good bioavailability and absorption through their possible oral administration. To evaluate the absorption in each of the compounds, the percentage of absorption (% ABS) (equation 1) was calculated using the calculated values of TPSA in each of the compounds [19,20]:

% ABS = 109 − (0.345 ×TPSA)

### 2.9. Calculation of Risk Toxicity

The Osiris Data Warrior computational tool was used to determine the toxicological behavior of the compounds extracted from *Blechnum*. The toxicological risks that were evaluated were mutagenicity, tumorigenicity, irritation and reproductive effect [19]. The toxicological comparison of the compounds extracted from *Blechnum* with known inhibitors of cholinesterases was made.

### 2.10. Docking Simulations

The crystallographic structures of the enzymes acetylcholinesterase from *Torpedo ca- lifornica* (TcAChE; PDBID: code 1DX6 [21]) and human butyrylcholinesterase (hBuChE; PDBID: code 4BDS [22]) were downloaded from the RCSB PDB protein data bank [23]. For the optimizations of the enzymes, the UCSF Chimera software (v1.16, San Francisco, CA, USA) was used, the water molecules and the ligands of the catalytic sites were removed. Polar hydrogen atoms added at pH = 7.4. The appropriate ionization states were considered for each of the amino acid residues, both basic and acidic. The centroid of the residue was chosen based on the putative catalytic site in each of the enzymes considering the known catalytic amino acids: Ser200 for acetylcholinesterase (TcAChE) [24,25] and Ser198 for butyrylcholinesterase (hBuChE) [26,27]. The creation of the two-dimensional structures of the ligands was carried out in the ChemDraw 8.0 software (PerkinElmer Informatics, Waltham, MA, USA). Subsequently, the two-dimensional structures were imported into the Avogadro software (https://avogadro.cc, accessed on 25 January 2023) where the geometric optimization and minimization of the molecules was carried out using MMFF94 as a force field function. Molecular docking was performed using the respective rigid crystallographic enzyme structures and the flexible ligands whose torsion angles were identified (for 10 independent urns per ligand). Targeted coupling was performed using the UCSF Chimera program [28,29] taking the reference inhibitor galantamine for acetylcholinesterase and butyrylcholinesterase as the catalytic site. Polar hydrogens and Gasteiger partial charges were added for the docking process; a grid chart was created using the Autodock Vina tools in the UCSF Chimera software. The results obtained from molecular docking were analyzed and visualized in the Discovery Studio Visualizer software [30]. Once the molecular docking was done, the best conformation at the catalytic site was analyzed by evaluating the best hydrogen bonding interactions or π interactions, including the free ligand binding energy (kcal/mol) [29,31].

### 2.11. Statistical Analysis 

Three measurements were obtained from each sample solution and the results were expressed as mean values ± standard deviations (SD) using Microsoft Excel 2019 (Microsoft Corporation, Redmond, WA, USA). Using GraphPad Prism 8 software (Corporation, La Jolla, CA, USA), a one-way analysis of variance was performed using Tukey’s test (*p* ˂ 0.05).

## 3. Results and Discussion

### 3.1. Metabolite Profiling of Fern Extracts 

#### 3.1.1. Chromatographic Analysis of *Blechnum chilense*

**Figure 3:**
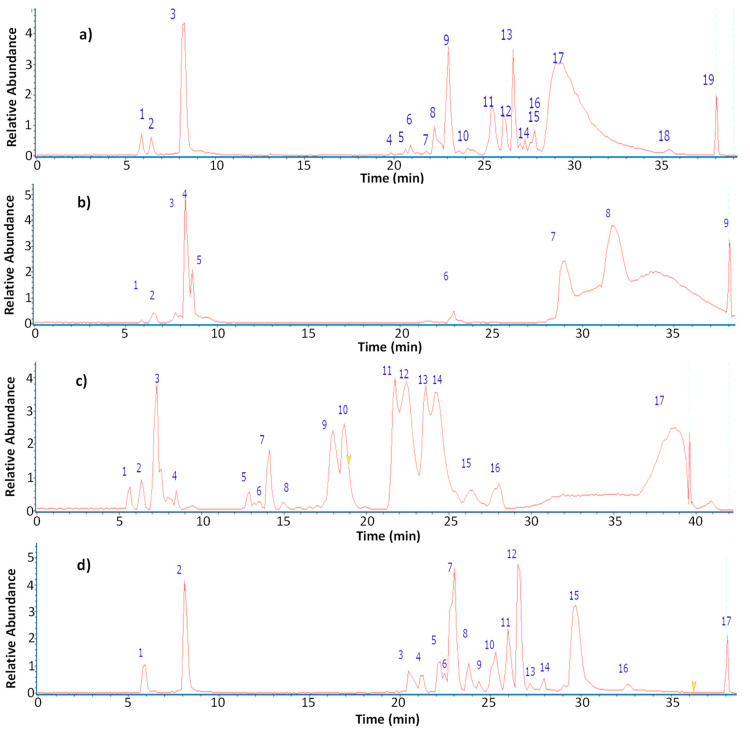
UHPLC-MS Chromatograms (**a**) Blechnum chilense; (**b**) Blechnum hastatum; (**c**) Blechnum magellanicum; (**d**) Blechnum penna-marina.

Chemical profiles of the freeze-dried extract of *B. chilense* were obtained by high-resolution mass spectrometry analysis (UHPLC-MS). Nineteen peaks were tentatively identified using the negative mode of the technique (Figure 3a) and corresponded to metabolites of organic acids, aromatics, carbohydrates and flavonoids (Table 1).

##### Organic Acid

Peak 2 was tentatively identified as 2,3,4,5-tetra-*O*-acetylhexaric acid (C_14_H_18_O_2_).

##### Aromatic Derivates

Peak 4 was tentatively identified as di-coumaroylquinic acid (C_25_H_23_O_12_), and peak 5 was tentatively identified as an isomer of di-coumaroylquinic acid, with a molecular anion *m/z* 515.1077 and with diagnostic peaks at *m*/*z* 353.0793—191.0524 and 353.0793—191.0514, respectively. Peak 6, with an [M-H]^−^ ion at *m*/*z* 359.07137 and diagnostic peaks at *m*/*z* 271.0902 and 179.0318, was tentatively identified as 5,7,4’-trihydroxy-3,8,3’-trymethoxyflavone (C_18_H_15_O_8_), and peak 7 was tentatively identified as 3,5-di-*O*-caffeoylquinic acid (C_25_H_24_O_12_). Peak 8 was tentatively identified as 3-*O*-caffeoylshikimic acid (C_9_H_23_O_12_), with a molecular anion *m/z* 335.0713 and with diagnostic peaks at *m*/*z* 296.04122 and 179.0316, and peak 10 was tentatively identified as an isomer of 3-*O*-caffeoylshikimic acid. Peak 9 was tentatively identified as 5-*O*-caffeoylshikimic acid (C_9_H_23_O_12_), with a molecular anion *m/z* 335.0713 and with diagnostic peaks at *m*/*z* 296.04122 and 179.0316, and peak 11 was tentatively identified as an isomer of 5-*O*-caffeoylshikimic acid. Peak 14 was tentatively identified as daphnorin (C_25_H_22_O_12_), and peak 15 was tentatively identified as cirsimaritin (C_17_H_13_O_6_), with an [M-H]^−^ ion at *m*/*z* 313.0717 and diagnostic peaks at *m*/*z* 271.0798, 627.14120 and 270.0795. Peak 19 corresponded to Na formiate (C_4_H_2_O_4_) as the internal standard.

##### Carbohydrates

Peak 3 was tentatively identified as 3-*O*-caffeoylglucose (C_15_H_18_O_9_), and peak 16 was tentatively identified as 1-*O*,7-*O*-digalloyl-D-sedoheptulose C_21_H_21_O_15_).

##### Flavonoids

Peak 12, with a molecular anion at *m*/*z* 359.0713, was tentatively identified as irigenin (C_18_H_16_O_8_), with diagnostic peaks at *m*/*z* 271.0902 and 179.0318. Peak 13 was tentatively identified as persicogenin 3’-*O*-glucoside (C_23_H_25_O_11_), with a molecular anion at *m*/*z* 477.1307 and diagnostic peaks at *m*/*z* 269.0752 and 159.0457. Peak 17 was tentatively identified as quercetin-3-*O*-acetate (C_17_H_11_O_8_), with an [M-H]^−^ ion at *m*/*z* 343.04097 and diagnostic peaks at *m*/*z* 299.0510 and 271.0551. Peak 18, with a molecular anion at *m*/*z* 299.0502, was tentatively identified as chrysoeriol (C_16_H_12_O_6_).

##### Unknown

Peak 1 was detected but not identified.

#### 3.1.2. Chromatographic Analysis of *Blechnum hastatum*

Chemical profiles of the freeze-dried extract of *B. hastatum* were obtained by high-resolution mass spectrometry analysis (UHPLC-MS). Nine peaks were tentatively identified using the negative mode of the technique (Figure 3b) and corresponded to metabolites of carbohydrates, aromatics and flavonoids (Table 2).

##### Carbohydrates

Peak 1, with a molecular anion at *m*/*z* 503.1401, was tentatively identified as 6-*O*-caffeoylsucrose (C_21_H_28_O_14_); peak 2 was tentatively identified as glyvenol (C_29_H_33_O_6_) with a molecular anion at 476.22631; peak 3 was tentatively identified as 3-[(*E*)-3-[5-(2-methoxycarbonylphenyl)furan-2-yl]prop-2-enoyl]-6-methyl-4-oxopyran-2-olate, with an [M-H]^−^ ion at *m*/*z* 378.0686 and diagnostic peaks at *m*/*z* 341.0946 and 191.0498; peak 8, with a molecular anion at *m*/*z* 403.2067, was tentatively identified as lauroside B (C_19_H_31_O_9_), with diagnostic peaks at *m*/*z* 321.2352 and 317.2018.

##### Aromatic Derivates

Peak 4 was tentatively identified as 3-*O*-caffeoylglucose (C_15_H_18_O_9_), and peak 5 was tentatively identified as 4-*O*-caffeoylglucose (C_15_H_18_O_9_), with a molecular anion *m/z* 341.0971 and with diagnostic peaks at *m*/*z* 193.0268 and 193.0265, respectively. Peak 7 was tentatively identified as cretanin (C_20_H_22_O_13_), with an [M-H]^−^ ion at *m*/*z* 469.0927 and diagnostic peaks at *m*/*z* 295.0435 and 335.0622. Peak 9 corresponded to Na formiate (C_4_H_2_O_4_) as the internal standard.

##### Flavonoids

Peak 6 was tentatively identified as acetylgenistin (C_23_H_21_O_11_), with an [M-H]^−^ ion at *m*/*z* 473.10306.

#### 3.1.3. Chromatographic Analysis of *Blechnum magellanicum*

Chemical profiles of the freeze-dried extract of *B. magellanicum* were obtained by high-resolution mass spectrometry analysis (UHPLC-MS). Seventeen peaks were tentatively identified using the negative mode of the technique (Figure 3c) and corresponded to metabolites of carbohydrates, aromatics and flavonoid (Table 3).

##### Aromatic Derivates

Peaks 5, 7, 8, 11, 12, 13, 14 and 17 were tentatively identified as glucose gallate (C_13_H_15_O_11_), phloroglucin-1-*O*-(6″-galloyl-glucoside) (C_19_H_19_O_12_), caffeoyl-hexoside malate (C_19_H_21_O_13_), 3-O-galloylmalic acid (C_9_H_23_O_12_), 2-*O*-galloylmalic acid (C_9_H_23_O_12_), 4-*O*-galloylmalic acid (C_9_H_23_O_12_), 3-*O*-caffeoyl-5-*O*-malonylquinic acid (C_19_H_19_O_12_) and galloyl citrate (C_13_H_11_O_11_), respectively.

##### Carbohydrates

Peak 2 was tentatively identified as 6-*O*-caffeoylsucrose (C_19_H_18_O_6_), with an [M-H]^−^ ion at *m*/*z* 503.1401; peak 6 was tentatively identified as methyl vanillate glucoside (C_15_H_19_O_9_), with a molecular anion *m/z* 343.1035; peak 9 was tentatively identified as D-galactose fragment (C_12_H_25_O_11_), with a molecular anion *m/z* 345.1402.

##### Flavonoids

Peak 3 was tentatively identified as zapotin (C_19_H_18_O_6_), with an [M-H]^−^ ion at *m*/*z* 341.0971.

##### Unknowns

Peaks 1, 4, 10, 15 and 16 were detected but not identified.

#### 3.1.4. Chromatographic Analysis of *Blechnum penna-marina*

Chemical profiles of the freeze-dried extract of *B. penna-marina* were obtained by high-resolution mass spectrometry analysis (UHPLC-MS). Seventeen peaks were tentatively identified using the negative mode of the technique (Figure 3d) and corresponded to metabolites of carbohydrates, aromatics and flavonoids (Table 4).

##### Aromatic Derivates

Peak 3, with an [M-H]^−^ ion at *m*/*z* 515.1077, was tentatively identified as di-coumaroylquinic acid (C_25_H_23_O_12_) and diagnostic peaks at *m*/*z* 353.0793 and 191.0524; peak 4 was tentatively identified as 3-*O*-galloylmalic acid (C_9_H_23_O_12_), with a molecular anion *m*/*z* 285.0252; peaks 5 and 6, with an [M-H]^−^ ion at *m*/*z* 335.0713 and diagnostic peaks at *m*/*z* 296.04122 and 179.0316, were tentatively identified as 3-*O*-caffeoylshikimic acid and 5-*O*-caffeoylshikimic acid; peak 7 was tentatively identified as 4-*O*-galloylmalic acid (C_9_H_23_O_12_), with a molecular anion 285.0252; peak 9 was tentatively identified as 3-*O*-caffeoyl-5-*O*-malonylquinic acid (C_19_H_19_O_12_), with an [M-H]^−^ ion at 439.0823; peak 11 was tentatively identified as theaflavin-3-gallate (C_36_H_27_O_16_), with a molecular anion *m*/*z* 715.1219; peak 13 was tentatively identified as 1-*O*,7-*O*-digalloyl-D-sedoheptulose (C_21_H_21_O_15_), with an [M-H]^−^ ion at *m*/*z* 513.1038; peak 14, with a molecular anion *m/z* 469.0927, was tentatively identified as cretanin (C_20_H_22_O_13_), and diagnostic peaks at *m*/*z* 295.0435 and 335.0622; peak 16 was tentatively identified as galloyl citrate (C_13_H_11_O_11_), with an [M-H]^−^ ion at *m*/*z* 343.0400. Peak 17 corresponded to Na formiate (C_4_H_2_O_4_) as the internal standard.

##### Carbohydrates

Peaks 1 and 2 were tentatively identified as 6-*O*-caffeoylsucrose (C_21_H_28_O_14_) and 3-*O*-caffeoylglucose (C_15_H_18_O_9_), respectively, and with an [M-H]^−^ ion at *m*/*z* 503.1401 and 341.1030, respectively.

##### Flavonoids

Peak 8 was tentatively identified as rutin (C_12_H_25_O_11_), with an [M-H]^−^ ion at *m*/*z* 609.1361. Peak 10 was tentatively identified as cirsimaritin (C_17_H_14_O_6_), with a molecular anion *m*/*z* 313.0658 and diagnostic peaks at *m*/*z* 271.0798 and 627.14120. Peak 15 was tentatively identified as chrysoeriol (C_16_H_12_O_6_), with an [M-H]^−^ ion at *m*/*z* 299.0502.

##### Unknowns

Peak 12 was detected but not identified.

Most of the secondary metabolites reported in *B. chilense, B. hastatum, B. magellanicum* and *B. penna-marina* species are grouped in phenolic compounds and organic acids that have also been reported in species such as *B. orientale, B. novae-zelandiae, B. occidentale* and *B. binervatum* [32,33,34,35,36]. In the four study species, the compounds neophytadiene, phytol (3,7,11,15-tetramethyl-2-hexadecen-1-ol), isophytol (3,7,11,15-tetramethyl-1-hexadecen-3-ol), ecdysone, 2-deoxyecdysone (2-deoxycrusteecdysone), ponasterone, shidasterone and β-sitosterol (stigmast-5-en-3-ol) have also been reported [35,37,38,39,40]. Flavonoid-like compounds present in species of the genus *Blechnum* are also shared by species of the genera *Pteris* and *Pteridium* of the family Pteridaceae [41]. These compounds in general exhibit diverse bioactive properties such as antimicrobial, anti-inflammatory, antioxidant and neuroprotective properties, which have been studied by in vitro and in vivo assays and represent a promising pharmacological resource [42,43,44].

### 3.2. Total Phenolic and Flavonoid Content and Antioxidant Activity

The values obtained for the content of phenolic compounds and flavonoids in the freeze-dried extracts of the four *Blechnum* species were high, with significant antioxidant activity (Table 5). The highest concentration of total phenols was found in the extract of *B. penna-marina* (88.846 ± 0.020 mg GAE/g), followed by *B. chilense* (34.078 ± 0.010 mg GAE/g); likewise, the flavonoid content stood out in *B. penna-marina* (128.662 ± 0.065 mg QE/g), followed by similar values in *B. chilense* (52.959 ± 0.055 mg QE/g) and *B. magellanicum* (52.408 ± 0.052 mg QE/g). As for the DPPH assay, the most efficient IC_50_ result was shown by *B. penna-marina* (41.818 ± 0.005 µg/mL), as well as higher values in the FRAP (3301.847 ± 1.050 µmol Trolox/g) and ORAC (2677.519 ± 0.096 µmol Trolox/g) tests, which support high antioxidant activity. 

The content of total phenols in *B. penna-marina* in grams of the dry plant is comparable with *B. occidentale* where values of 2095 GAE/g have been reported [45]; on the other hand, its ORAC and DPPH values are outstanding as those reported for the methanolic extract of *B. spicant* species with 2910.7 µmol Trolox/g and 11.42 µg/mL, respectively [46]. In addition, the results of *B. penna-marina* are moderately comparable with the species *Aristotelia chilensis* “maqui”, which represents a raw material endemic to Chile and is known as a standard among the rich sources of antioxidants and registers an ORAC value of 3900 to 29,600 µmol Trolox/g [47]. The antioxidant properties of the four study species are potent and comparable to the significant effects reported for *B. binervatum, B. brasiliense*, *B. occidentale* and *B. orientale* [33,35,48].

### 3.3. Enzyme Inhibitory Activity

The lyophilized extracts of the four *Blechnum* species showed variable cholinesterase activity, with a high potential for inhibition of the enzyme acetylcholinesterase (Table 6). For AChE the inhibition range was between 8.311 ± 0.028 to 12.252 ± 0.028 µg/mL, with better results in *B. hastatum* and *B. penna-marina* species. On the other hand, only the species *B. penna-marina* presented a significant inhibition of the BChE enzyme, with an IC_50_ of 27.151 ± 0.078 µg/mL; in the case of the species *B. chilense, B. hastatum* and *B. magellanicum* species, no inhibition was detected with the working concentrations, so it is recommended to increase the concentration above 25 µg/mL for dilutions in in vitro assays; however, with the molecular docking study, a high inhibition potential was found at the level of compounds present in the extracts of these species: 3-*O*-caffeoyl-5-*O*-malonylquinic acid, quercetin-3-*O*-acetate, chrysoeriol, cirsimaritin, 5-*O*-caffeoylshikimic acid, irigenin, cirsimaritin, zapotin and methyl vanillate glucoside. As for AChE, the reported inhibitory activity was better with an IC_50_ lower than 13 µg/mL, compared to reports in other species of the genus *Blechnum* [44]. Likewise, these results are comparable with the ferns species *Lemmaphyllum carnosum* (Polypodiaceae), which reports an IC_50_ for AChE of 16.6 µg/mL [49] and *Dryopteris erythrosora* [50]. In summary, the species *B. penna-marina* is positioned as a fern with a high potential for enzymatic inhibition in the genus *Blechnum*.

### 3.4. ADME Prediction and Toxicity Prediction

**Figure 4:**
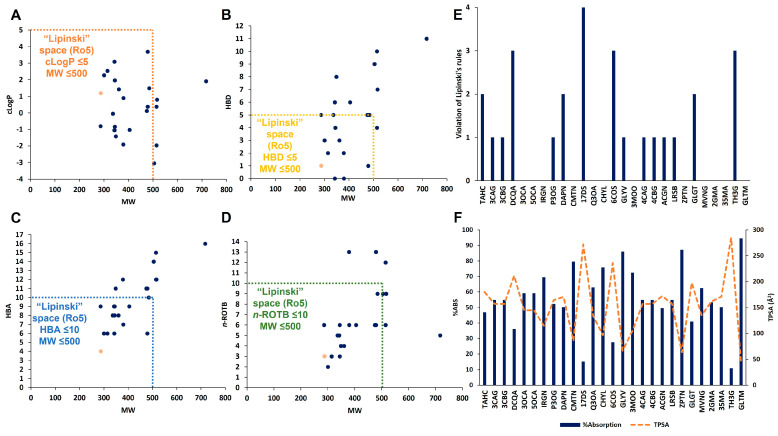
Pharmacokinetic properties of compounds present in the species of the genus *Blechnum* in comparison with the standard inhibitor galantamine on cholinesterases. (**A**) Plot of cLogP vs. molecular weight highlights all compounds that follow Lipinski’s “rule of five” (Lipinski space, orange box); (**B**) Plot of number of hydrogen bond donors vs. weight molecular weight highlights all compounds that follow Lipinski’s “rule of five” (Lipinski space, yellow box); (**C**) The plot of number of hydrogen bond acceptors versus molecular weight highlights all compounds that follow Lipinski’s “rule of five” (Lipinski space, blue box); (**D**) The plot of number of rotatable bonds against molecular weight highlights all compounds that follow Lipinski’s “rule of five” (Lipinski space, green box); (**E**) Number of violations of the compounds of the genus Blechnum; (**F**) Percentage of Absorption and TPSA values of the compounds of the genus *Blechnum*. Note: THC (2,3,4,5-tetra-*O*-acetylhexaric acid), 3CAG (3-*O*-caffeoyl-alpha-glucose), 3CBG (3-*O*-caffeoyl-beta-glucose), DCQA (3,5-di-*O*-caffeoylquinic acid), 3OCA (3-*O*-caffeoylshikimic acid), 5OCA (5-*O*-caffeoylshikimic acid), IRGN (irigenin), P3OG (persicogenin 3’-*O*-glucoside), DAPN (daphnorin), CMTN (cirsimaritin), 17DS (1-*O*,7-*O*-digalloyl-D-sedoheptulose), Q3OA (quercetin-3-*O*-acetate), CHYL (chrysoeriol), 6COS (6-caffeoyl sucrose), GLYV (glyvenol), 3MOO (3-[(E)-3-[5-(2-methoxycarbonylphenyl)furan-2-yl]prop-2-enoyl]-6-methyl-4-oxopyran-2-olate), 4CAG (4-*O*-caffeoyl-alfa-glucose), 4CBG (4-*O*-caffeoyl-beta-glucose), ACGN (acetylgenistin), LRSB (lauroside B), ZPTN (zapotin), CLGT (glucose gallate), MVNG (methyl vanillate glucoside), 2GMA (2-*O*-galloyl-L-malic acid), 35MA (3-*O*-caffeoyl-5-*O*-malonylquinic acid), TH3G (theaflavin-3-gallate), GLTM (galantamine).

The compounds obtained from the chemical characterization of the lyophilized extracts of *Blechnum* species were subjected to pharmacokinetic analysis comparing the results with galantamine, which is a known inhibitor for cholinesterases (TcAChe and hBuChE). A good drug candidate must exhibit a certain behavior that can be measurable by Lipinski’s “rule of five”; this rule mentions that a compound in preclinical studies must have a molecular weight (MW) not greater than 500 Da, have a number fewer than or equal to 10 rotating bonds, 10 or fewer acceptor hydrogen bonds, 5 or fewer hydrogen donor bonds and a cLog *p* value ≤ 5. Figure 4 shows the results obtained from the pharmacokinetic properties of the 26 compounds from the Blechnum species (THC (2,3,4,5-tetra-*O*-acetylhexaric acid), 3CAG (3-*O*-caffeoyl-alpha-glucose), 3CBG (3-*O*-caffeoyl-beta-glucose), DCQA (3,5-di-*O*-caffeoylquinic acid), 3OCA (3-*O*-caffeoylshikimic acid), 5OCA (5-*O*-caffeoylshikimic acid), IRGN (irigenin), P3OG (persicogenin 3’-*O*-glucoside), DAPN (daphnorin), CMTN (cirsimaritin), 17DS (1-*O*,7-*O*-digalloyl-D-sedoheptulose), Q3OA (quercetin-3-*O*-acetate), CHYL (chrysoeriol), 6COS (6-caffeoyl sucrose), GLYV (glyvenol), 3MOO (3-[(E)-3-[5-(2-methoxycarbonylphenyl)furan-2-yl]prop-2-enoyl]-6-methyl-4-oxopyran-2-olate), 4CAG (4-*O*-caffeoyl-alfa-glucose), 4CBG (4-*O*-caffeoyl-beta-glucose), ACGN (acetylgenistin), LRSB (lauroside B), ZPTN (zapotin), CLGT (glucose gallate), MVNG (methyl vanillate glucoside), 2GMA (2-*O*-galloyl-L-malic acid), 35MA (3-*O*-caffeoyl-5-*O*-malonylquinic acid), TH3G (theaflavin-3-gallate)); in addition, those compounds that do not comply with the Lipinski rule are shown (Figure 4E) and cannot be taken as possible cholinesterase inhibitor candidates. Figure 4A–D show that of the 26 compounds evaluated, 5 are outside the zone of a molecular mass of less than 500 Da and a cLogP less than 5 (Figure 4A); 8 compounds do not enter the list, have a zone of a molecular mass less than 500 Da and have a number fewer than or equal to 5 hydrogen bond donors (Figure 4B); 8 compounds do not enter the zone of a molecular mass less than or equal to 500 Da and have fewer than or equal to 10 hydrogen acceptor bonds (Figure 4C); and lastly 6 compounds do not enter the area of having fewer than or equal to 10 rotatable bonds and a molecular mass less than or equal to 500 Da (Figure 4D). 

In summary, of the 26 compounds submitted to the pharmacokinetic evaluation, 19 met the criteria of presenting one or no violation of the Lipinski rule (Figure 4E). The 19 compounds that presented one or no violation of the Lipinski rule underwent the prediction of the toxicological analysis using the Osiris Data Warrior computational tool. Subsequently, the compounds that did not violate any Lipinski rule and did not present any toxicological risk were analyzed by molecular coupling with the crystallographic structures of cholinesterases to observe which of these compounds could be a candidate inhibitor of these enzymes.

**Figure 5:**
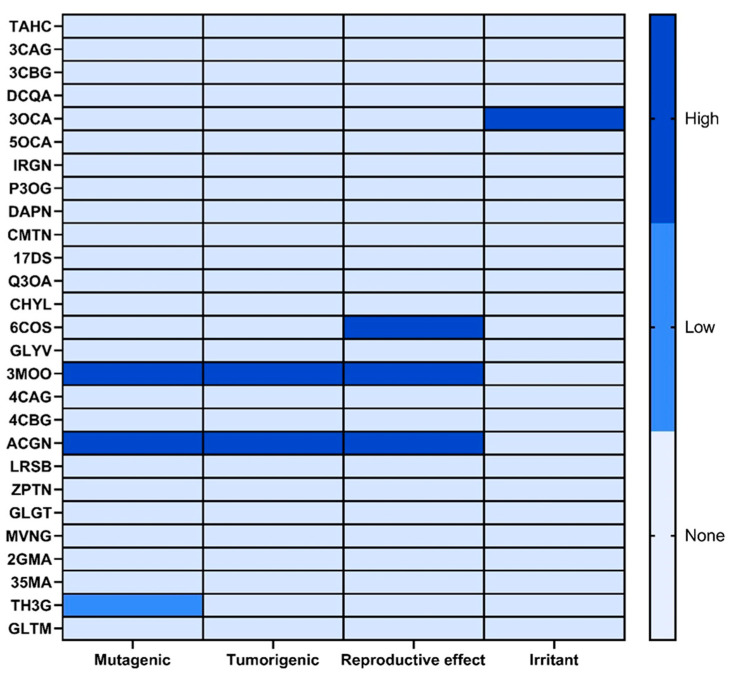
Calculation of toxicity risks of compounds present in the species of the genus *Blechnum* in comparison with the standard inhibitor galantamine on cholinesterases. Note: THC (2,3,4,5-tetra-*O*-acetylhexaric acid), 3CAG (3-*O*-caffeoyl-alpha-glucose), 3CBG (3-*O*-caffeoyl-beta-glucose), DCQA (3,5-di-*O*-caffeoylquinic acid), 3OCA (3-*O*-caffeoylshikimic acid), 5OCA (5-*O*-caffeoylshikimic acid), IRGN (irigenin), P3OG (persicogenin 3’-*O*-glucoside), DAPN (daphnorin), CMTN (cirsimaritin), 17DS (1-*O*,7-*O*-digalloyl-D-sedoheptulose), Q3OA (quercetin-3-*O*-acetate), CHYL (chrysoeriol), 6COS (6-caffeoyl sucrose), GLYV (glyvenol), 3MOO (3-[(E)-3-[5-(2-methoxycarbonylphenyl)furan-2-yl]prop-2-enoyl]-6-methyl-4-oxopyran-2-olate), 4CAG (4-*O*-caffeoyl-alfa-glucose), 4CBG (4-*O*-caffeoyl-beta-glucose), ACGN (acetylgenistin), LRSB (lauroside B), ZPTN (zapotin), CLGT (glucose gallate), MVNG (methyl vanillate glucoside), 2GMA (2-*O*-galloyl-L-malic acid), 35MA (3-*O*-caffeoyl-5-*O*-malonylquinic acid), TH3G (theaflavin-3-gallate), GLTM (galantamine).

The bioavailability of the 26 species compounds from *Blechnum* was tested and evaluated by TPSA assay. TPSA is highly involved with the passive transport that can take place in cell membranes. The TPSA values allowed us to calculate the absorption of the compounds using equation 1. In Figure 4F, the compounds that presented a greater absorption were IRGN (69.43%), CMTN (79.60%), CHYL (75.80%), GLYV (86.01%), 3MOO (72.60%) and ZPTN (87.19%); however, these compounds did not present higher values than the reference inhibitor galantamine (GLMN) because the TPSA value in galantamine was considerably higher compared to these compounds. Although the 3MOO compound presented an adequate absorption percentage, it was discarded for the molecular coupling analyzes because it presented high toxicity values (Figure 5).

The pharmacodynamic (toxicological) properties of the compounds were evaluated using the Osiris Data Warrior computational tool; the risks of toxicity that were evaluated were irritation, reproductive toxicity, tumorigenicity and mutagenicity (Figure 5). The results showed that the 3OCA compound, although it did not present any violation in the evaluation of the pharmacokinetic properties, presented a high risk of irritability because the chemical fragment corresponding to the ester with the double bond is the one that confers this irritant property (Figure 5). The 6COS compound presented a high risk of toxicity due to the chirality that it presents in its chemical structure, mainly in the fraction of hexopyranoside and furanosyl; in addition, it does not comply with the violations allowed in the pharmacokinetic properties (Figure 5). The compounds 3MOO and ACGN presented a similar behavior regarding the risks of toxicity; both presented high risks of mutagenicity, tumorigenicity and reproductive effect. Both compounds presented good pharmacokinetic properties; however, various fragments in their chemical structure in both compounds are what give them high toxicity (Figure 5). The last compound that presented a risk of toxicity was TH3G; this presented a low risk of mutagenicity because the ring with conjugated double bond in its structure is what gives it this low mutagenicity (Figure 5).

### 3.5. Docking Results

Of the 26 compounds obtained from the genus *Blechnum*, those compounds that did not present any violation of the pharmacokinetic properties and that did not present any risk of toxicity were selected, thus obtaining 9 compounds (5OCA, IRGN, CMTN, Q3OA, CHYL, ZPTN, MVNG, 2GMA and 35MA) that were used for molecular docking assays. These nine compounds were analyzed for molecular protein interactions with the main amino acid residues involved in the inhibition of acetylcholinesterase and butyrylcholinesterase using the well-known cholinesterase inhibitor (galantamine) as a reference in both cases. The best binding energies for each of the ligands were expressed in kcal/mol and compared with the binding energies of the galantamine inhibitor.

#### 3.5.1. Acetylcholinesterase (TcAChE) Docking Results

**Figure 6:**
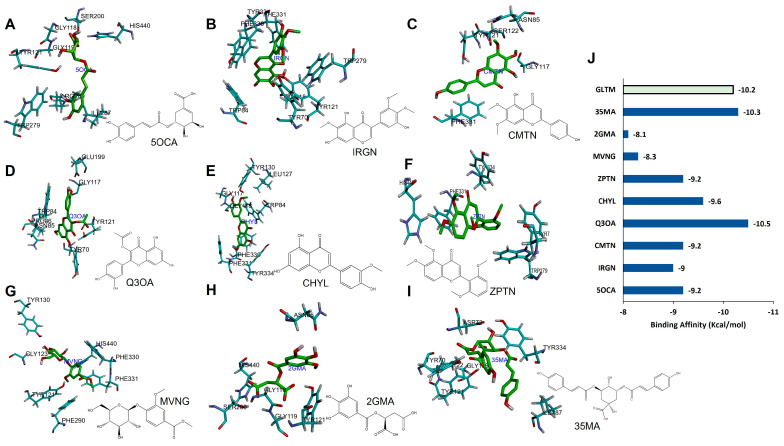
Molecular docking results from *Blechnum* extracts on the *Torpedo californica* acetylcholinesterase (TcAChE) catalytic site. (**A**) Docking results of 5OCA on TcAChE catalytic site; (**B**) Docking results of IRGN on TcAChE catalytic site; (**C**) Docking results of CMTN on TcAChE catalytic site; (**D**) Docking results of Q3OA on TcAChE catalytic site; (**E**) Docking results of CHYL on TcAChE catalytic site; (**F**) Docking results of ZPTN on TcAChE catalytic site; (**G**) Docking results of MVNG on TcAChE catalytic site; (**H**) Docking results of 2GMA on TcAChE catalytic site; (**I**) Docking results of 35MA on TcAChE catalytic site; (**J**) Binding affinities resulting from molecular docking experiments of the selected compounds in the extracts of *Blechnum*, together with the standard inhibitor galantamine on acetylcholinesterase (TcAChE). Note: 5OCA (5-*O*-caffeoylshikimic acid), IRGN (irigenin), CMTN (cirsimaritin), Q3OA (quercetin-3-*O*-acetate), CHYL (chrysoeriol), ZPTN (zapotin), MVNG (methyl vanillate glucoside), 2GMA (2-*O*-galloyl-L-malic acid), 35MA (3-*O*-caffeoyl-5-*O*-malonylquinic acid), GLTM (galantamine).

**Figure 7:**
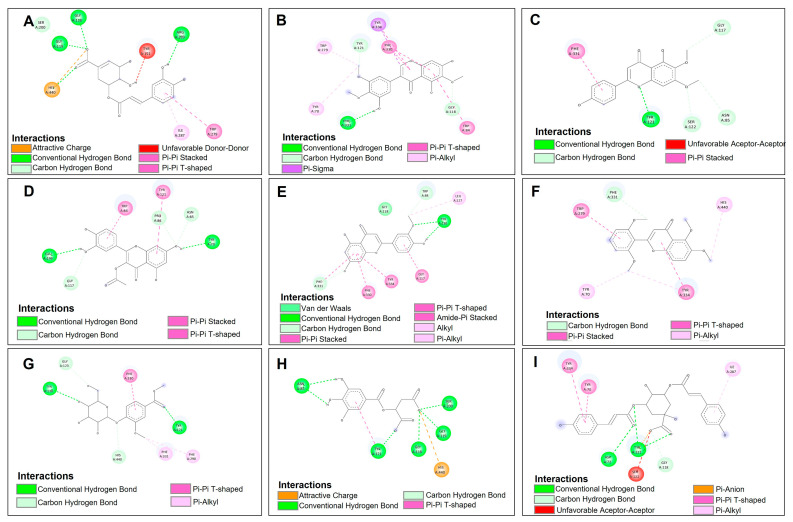
Molecular interactions of the compounds present in the species of *Blechnum* on the *Torpedo californica* acetylcholinesterase (TcAChE) catalytic site. (**A**) Molecular interactions of 5OCA; (**B**) Molecular interactions of IRGN; (**C**) Molecular interactions of CMTN; (**D**) Molecular interactions of Q3OA; (**E**) Molecular interactions of CHYL; (**F**) Molecular interactions of ZPTN; (**G**) Molecular interactions of MVNG; (**H**) Molecular interactions of 2GMA; (**I**) Molecular interactions of 35MA. Note: 5OCA (5-*O*-caffeoylshikimic acid), IRGN (irigenin), CMTN (cirsimaritin), Q3OA (quercetin-3-*O* acetate), CHYL (chrysoeriol), ZPTN (zapotin), MVNG (methyl vanillate glucoside), 2GMA (2-*O*-galloyl-L-malic acid), 35MA (3-*O*-caffeoyl-5-*O*-malonylquinic acid).

The molecular coupling of the compounds (5OCA, IRGN, CMTN, Q3OA, CHYL, ZPTN, MVNG, 2GMA and 35MA) present in the species of the genus *Blechnum* was performed and analyzed. These compounds (5OCA, IRGN, CMTN, Q3OA, CHYL, ZPTN, MVNG, 2GMA and 35MA) were chosen to carry out the molecular coupling with the acetylcholinesterase enzyme since they did not present any risk of toxicity and presented good behavior in pharmacokinetic properties. Compounds were compared to the known acetylcholinesterase inhibitor (galantamine) (Figure 6J). The results showed that Q3OA was the compound that presented a higher affinity (−10.5 kcal/mol) in the catalytic site of acetylcholinesterase in comparison with the evaluated compounds and with the known acetylcholinesterase inhibitor galantamine (Figure 6D,J). This behavior is mainly because I present two strong hydrogen bond type interactions with residues Tyr70 and Glu199; in addition to that, I present 3 carbon hydrogen bond type interactions with residues Asn85, Pro86 and Gly117 that confer stability to the conformation of Q3OA at the catalytic site of acetylcholinesterase (Figure 7D).

One of the compounds that presented a behavior like that of the inhibitor galantamine was the compound 35MA since it presented an affinity of −10.3 kcal/mol (Figure 6J). This good behavior in the catalytic site of acetylcholinesterase is because it presented 3 strong hydrogen bond interactions with the Asp72 residues and two with Tyr121 (Figure 6I and Figure 7I); these interactions conferred an important stability on the catalytic site of acetylcholinesterase inhibition (Figure 6I). Similarly, it was observed that the 35MA compound presented an interaction of the carbon-hydrogen bond type with the residue Gly118 (Figure 7I). This compound presented a strong electrostatic interaction between the carboxylate group of its structure and the Tyr121 residue, which allows the 35MA conformation to be more stable in this catalytic site due to the electrostatic attraction with this residue (Figure 7I).

The compounds CHYL, ZPTN, CMTN, 5OCA and IRGN presented similar behaviors in the binding affinity (−9.6, −9.2, −9.2, −9.2 and −9.0, respectively) (Figure 6J). The interactions presented by these compounds were very similar since they presented hydrogen bond type interactions, carbon hydrogen bonds, π−π interactions and alkyl interactions (Figure 7B,C,E,F). For these reasons, the conformations within the catalytic site of these compounds were very similar (Figure 6B,C,E,F), which led to the fact that they presented a similar binding affinity at the acetylcholinesterase catalytic site.

The compounds that presented the lowest binding affinities were 2GMA and MVNG (−8.1 and −8.3 kcal/mol, respectively) (Figure 6J). The MVNG compound presented a higher affinity than the 2GMA compound because it presented six strong hydrogen bond type interactions with the Asn85, Tyr121, Gly118, Gly119 and Ser200 residues (Figure 7H). It should be noted that although the MVNG compound presented a lower affinity than the other compounds to be evaluated, it presented an important interaction with the Ser200 residue, which is the one that is directly involved in the inhibition of acetylcholinesterase. The reason that MVNG presents a lower affinity in the catalytic site may be due to the great influence that rotatable bonds have in its structure, which allows its stability in the catalytic site of the enzyme to decrease considerably (Figure 6H and Figure 7H).

#### 3.5.2. Butyrylcholinesterase (BChE) Docking Results

**Figure 8:**
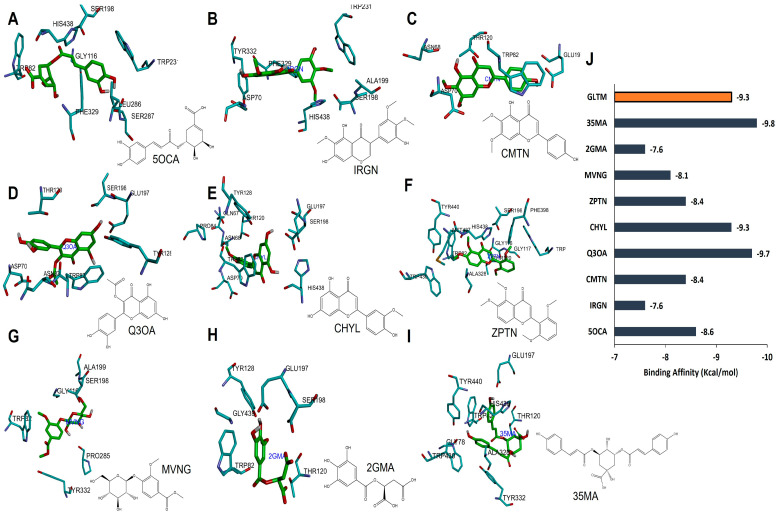
Molecular docking results from *Blechnum* extracts on the human butyrylcholinesterase (hBuChE) catalytic site. (**A**) Docking results of 5OCA on TcAChE catalytic site; (**B**) Docking results of IRGN on TcAChE catalytic site; (**C**) Docking results of CMTN on TcAChE catalytic site; (**D**) Docking results of Q3OA on TcAChE catalytic site; (**E**) Docking results of CHYL on TcAChE catalytic site; (**F**) Docking results of ZPTN on TcAChE catalytic site; (**G**) Docking results of MVNG on TcAChE catalytic site; (**H**) Docking results of 2GMA on TcAChE catalytic site; (**I**) Docking results of 35MA on TcAChE catalytic site; (**J**) Binding affinities resulting from molecular docking experiments of the selected compounds in the extracts of Blechnum, together with the standard inhibitor galantamine on acetylcholinesterase (TcAChE). Note: 5OCA (5-*O*-caffeoylshikimic acid), IRGN (irigenin), CMTN (cirsimaritin), Q3OA (quercetin-3-*O*-acetate), CHYL (chrysoeriol), ZPTN (zapotin), MVNG (methyl vanillate glucoside), 2GMA (2-*O*-galloyl-L-malic acid), 35MA (3-*O*-caffeoyl-5-*O*-malonylquinic acid), GLTM (galantamine).

**Figure 9:**
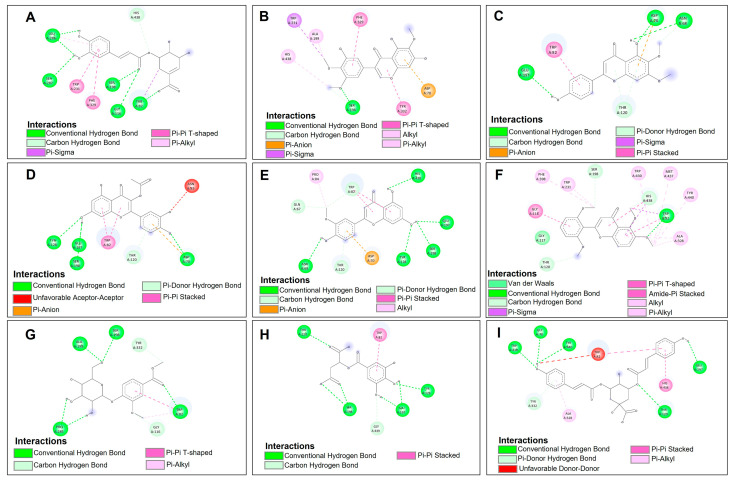
Molecular interactions of the compounds present in the species of *Blechnum* on the human butyrylcholinesterase (hBuChE) catalytic site. (**A**) Molecular interactions of 5OCA; (**B**) Molecular interactions of IRGN; (**C**) Molecular interactions of CMTN; (**D**) Molecular interactions of Q3OA; (**E**) Molecular interactions of CHYL; (**F**) Molecular interactions of ZPTN; (**G**) Molecular interactions of MVNG; (**H**) Molecular interactions of 2GMA; (**I**) Molecular interactions of 35MA. Note: 5OCA (5-*O*-caffeoylshikimic acid), IRGN (irigenin), CMTN (cirsimaritin), Q3OA (quercetin-3-*O*-acetate), CHYL (chrysoeriol), ZPTN (zapotin), MVNG (methyl vanillate glucoside), 2GMA (2-*O*-galloyl-L-malic acid), 35MA (3-*O*-caffeoyl-5-*O*-malonylquinic acid).

The results of the molecular coupling between the nine selected compounds from the extract of the Blechnum genus are shown in Figure 8. The molecular couplings showed that the compounds 35MA and Q3OA presented a higher affinity (−9.8 and −9.7 kcal/mol, respectively) than the reference inhibitor galantamine (−9.3 kcal/mol) (Figure 8J). These behaviors are mainly because both compounds presented strong hydrogen bond and π-anion type interactions (Figure 9D,I). Compound Q3OA presented a slightly lower affinity compared to compound 35MA because it presented unfavorable acceptor interactions between the Asn83 residue and the hydroxyl group of the aromatic ring of its structure (Figure 9D). Compound 35MA showed six strong hydrogen bonding interactions with residues Gly78, Thr120, Glu197, Trp430 and Tyr440, which give it high stability at the catalytic site of butyrylcholinesterase (hBuChE) (Figure 9I). Figure 8E,J and Figure 9E show the interactions and binding affinity of the CHYL compound, which presented a behavior like the known inhibitor galantamine. This behavior is mainly because the CHYL compound presented five strong hydrogen bond interactions with the residues Asn68, Tyr128, Glu197, Ser198 and His438 (Figure 9E). The interaction that occurred between the hydrogen of the aromatic hydroxyl group with the residue Ser198 makes it a good candidate inhibitor of butyrylcholinesterase because this residue (Ser198) participates directly in the inhibition of this enzyme, allowing the CHYL compound to suitably accommodate itself in the catalytic site of this enzyme (Figure 9E).

Other compounds that presented good binding affinities were the compounds 5OCA, CMTN, ZPTN and MVNG (−8.6, −8.4, −8.4 and −8.1 kcal/mol, respectively) (Figure 8J). The 5OCA compound was the one that presented a slightly higher binding affinity compared to the CMTN, ZPTN and MVNG compounds (Figure 8J) because it presented five hydrogen bond interactions with residues Trp82, Gly116, Ser198, Ser287 and Leu286 (Figure 8A and Figure 9A). There was an interaction with the Ser198 residue which, as previously mentioned, is involved in the inhibition of butyrylcholinesterase (Figure 8A and Figure 9A). Also presented were π-sigma interactions between the Trp82 residue and the methylene of cyclohexene; these interactions allow an important stability in the catalytic site of butyrylcholinesterase which, together with the hydrogen bonds, confers a greater binding affinity at the catalytic site (Figure 9A). Compounds 2GMA and IRGN presented the lowest binding affinities (both −7.6 kcal/mol), although they presented important interactions with the Ser198 residue (Figure 8B,H and Figure 9B,H). However, the rotatable bonds that these compounds present allow the dynamics of the molecule to be considerable, which prevents it from stabilizing properly in the catalytic site of the enzyme; since being molecules with several functional groups in their structure, these they confer a greater volume when they stabilize at the binding site (Figure 8B,H). However, the interactions shown by these two compounds can be treated as good butyrylcholinesterase inhibitor candidates.

## 4. Conclusions

The four studied species of the genus *Blechnum* present a variable abundance and heterogeneity in secondary metabolites, which are properly identified by the UHPLC-ESI-QTOF-MS method. The extracts indicate a high antioxidant activity, especially attributable to the content of phenolic compounds. The enzymatic activity of the extracts and compounds studied by molecular docking show a favorable inhibition on cholinesterase enzymes, especially in AChE, which demonstrates a potential benefit in alternative therapies and/or adjuvants for the treatment of neurodegenerative diseases such as Alzheimer’s disease. In all chemical and biological studies proposed in the future on these species of the genus *Blechnum*, the sustainability of the raw material in the different habitats where they are found should be considered, with the objective of not altering their presence, function and preservation in the ecosystem.

## Figures and Tables

**Table 1:** Identification of metabolites in *Blechnum chilense* by UHPLC-ESI-QTOF-MS.

Peak	Tentative Identification	[M-H]^−^	Retention Time (min.)	Theoretical Mass (*m/z*)	Measured Mass (*m/z*)	Accuracy (ppm)	Metabolite Type	MS Ions (ppm)
1	Unknown	C_4_HO_15_	5.90	299.93209	288.9299	2.0	-	272.9528
2	2,3,4,5-Tetra-*O*-acetylhexaric acid	C_14_H_18_O_2_	6.43	377.0725	377.0796	−6.0	OA	341.10414
3	3-*O*-Caffeoylglucose	C_15_H_18_O_9_	8.1	341.1030	341.1041	−6.9	CH	191.0513
4	Di-coumaroylquinic acid	C_25_H_23_O_12_	20.1	515.1077	515.1076	−3.2	A	353.0793, 191.0524
5	Di-coumaroylquinic acid (isomer)	C_25_H_23_O_12_	20.32	515.1077	515.1087	−3.2	A	353.0793, 191.0514
6	5,7,4’-Trihydroxy-3,8,3’-trymethoxyflavone	C_18_H_15_O_8_	20.88	359.07137	359.07024	2.5	A	271.0902, 179.0318
7	3,5-Di-*O*-caffeoylquinic acid	C_25_H_24_O_12_	20.94	515.1401	515.1407	1.12	A	191.0522
8	3-*O*-Caffeoylshikimic acid	C_9_H_23_O_12_	22.11	335.0713	335.0705	0.2	A	296.0412, 179.0316
9	5-*O*-Caffeoylshikimic acid	C_9_H_23_O_12_	22.98	335.0713	335.0708	0.2	A	296.0412, 179.0316
10	3-*O*-Caffeoylshikimic acid (isomer)	C_16_H_15_O_8_	24.27	335.0713	335.0719	0.6	A	296.0412, 179.0316
11	5. -*O*-Caffeoylshikimic acid (isomer)	C_16_H_15_O_8_	25.25	335.0713	335.0714	−7.2	A	296.0412, 179.0321
12	Irigenin	C_18_H_16_O_8_	26.32	359.0713	359.0702	−3.1	F	271.0902, 179.0318
13	Persicogenin 3’-*O*-glucoside	C_23_H_25_O_11_	26.92	477.1307	477.1402	−19.2	F	269.0752, 159.0457
14	Daphnorin	C_25_H_22_O_12_	27.20	513.1038	513.0958	−15.1	A	-
15	Cirsimaritin	C_17_H_13_O_6_	27.32	313.0717	313.0662	7.8	A	271.0798, 627.14120 (2M-H), 270.0795
16	1-*O*,7-*O*-Digalloyl-D-sedoheptulose	C_21_H_21_O_15_	28.21	513.1038	513.0963	8.2	CH	295.0549
17	Quercetin-3-*O*-acetate	C_17_H_11_O_8_	29.38	343.04097	343.0400	−14.4	F	299.0510, 271.0551
18	Chrysoeriol	C_16_H_12_O_6_	29.75	299.0502	299.0520	4.2	F	271.0550
19	Na formiate (internal standard)	C_4_H_2_O_4_	38.6	112.9829	112.9856	3.1	A	-

OA = organic acid; CH = carbohydrates; A = aromatic; F = flavonoid.

**Table 2:** Identification of metabolites in *Blechnum hastatum* by UHPLC-ESI-QTOF-MS.

Peak	Tentative Identification	[M-H]^−^	Retention Time (min.)	Theoretical Mass (*m/z*)	Measured Mass (*m/z*)	Accuracy (ppm)	Metabolite Type	MS Ions (ppm)
1	6-*O*-Caffeoylsucrose	C_21_H_28_O_14_	7.30	503.1401	503.14063	2.0	CH	425.0534
2	Glyvenol	C_29_H_33_O_6_	6.3	476.22631	476.22044	3.75	CH	-
3	3-[(*E*)-3-[5-(2-methoxycarbonylphenyl)furan-2-yl]prop-2-enoyl]-6-methyl-4-oxopyran-2-olate	C_28_H_10_O_2_	7.8	378.0686	378.0682	−0.95	CH	341.0946, 191.0498
4	3-*O*-Caffeoylglucose	C_15_H_18_O_9_	8.2	341.0971	341.0936	−10.2	A	193.0268
5	4-*O*-Caffeoylglucose	C_15_H_18_O_9_	8.9	341.0971	341.0932	16.0	A	193.0265
6	Acetylgenistin	C_23_H_21_O_11_	22.8	473.10306	473.10455	−9.7	F	279.0953
7	Cretanin	C_20_H_22_O_13_	28.94	469.0927	469.0928	3.0	A	295.0435, 335.0622
8	Lauroside B	C_19_H_31_O_9_	32.12	403.2067	403.1973	−9.7	CH	321.2352, 317.2018
9	Na formiate (internal standard)	C_4_H_2_O_4_	38.1	112.9829	112.9856	3.1	A	-

CH = carbohydrate; A = aromatic; F = flavonoid.

**Table 3:** Identification of metabolites in *Blechnum magellanicum* by UHPLC-ESI-QTOF-MS.

Peak	Tentative Identification	[M-H]^−^	Retention Time (min.)	Theoretical Mass (*m/z*)	Measured Mass (*m/z*)	Accuracy (ppm)	Metabolite Type	MS Ions (ppm)
1	Unknown	C_4_H_2_O_14_	5.70	273.9450	273.94790	10.5	-	174.9463
2	6-*O*-Caffeoylsucrose	C_19_H_18_O_6_	7.30	503.1401	503.14063	2.0	CH	425.0534
3	Zapotin	C_19_H_18_O_6_	8.1	341.0971	341.0966	−1.45	F	191.0513
4	Unknown	C_11_H_17_O_12_	12.93	341.0740	341.0722	4.37	-	179.0273, 679.1807 (2M-H)
5	Glucose gallate	C_13_H_15_O_11_	14.13	347.0619	347.0631	3.34	A	193.0419
6	Methyl vanillate glucoside	C_15_H_19_O_9_	17.1	343.1035	343.1023	3.3	CH	283.2497
7	Phloroglucin-1-*O*-(6″-galloyl-glucoside)	C_19_H_19_O_12_	18.0	439.0882	439.0899	−3.8	A	-
8	Caffeoyl-hexoside malate	C_19_H_21_O_13_	18.7	457.0988	457.0996	−1.7	A	-
9	D-Galactose fragment	C_12_H_25_O_11_	19.9	345.1402	345.1397	1.7	CH	-
10	Unknown	C_15_H_21_O_15_	21.8	441.0886	441.0900	−3.2	-	-
11	3-*O*-Galloylmalic acid	C_9_H_23_O_12_	22.14	285.0252	285.0282	12.3	A	241.9917
12	2-*O*-Galloylmalic acid	C_9_H_23_O_12_	22.27	285.0252	285.0274	10.8	A	240.9913
13	4-*O*-Galloylmalic acid	C_9_H_23_O_12_	22.27	285.0252	285.0284	7.8	A	240.9913
14	3-*O*-Caffeoyl-5-*O*-malonylquinic acid	C_19_H_19_O_12_	23.91	439.0823	439.0836	3.0	A	341.1264
15	Unknown	C_17_H_35_O_13_	28.1	447.2083	447.2075	1.7	-	-
16	Unknown	C_15_H_31_O_7_	34.5	323.2075	323.2079	−1.1	-	-
17	Galloyl citrate	C_13_H_11_O_11_	38.6	343.0400	343.0358	−12.4	A	299.0464

CH = carbohydrate; F = flavonoid; A = aromatic.

**Table 4:** Identification of metabolites in *Blechnum penna-marina* by UHPLC-ESI-QTOF-MS.

Peak	Tentative Identification	[M-H]^−^	Retention Time (min.)	Theoretical Mass (*m/z*)	Measured Mass (*m/z*)	Accuracy (ppm)	Metabolite Type	MS Ions (ppm)
1	6-*O*-Caffeoylsucrose	C_21_H_28_O_14_	7.30	503.1401	503.14063	2.0	CH	425.0534
2	3-*O*-Caffeoylglucose	C_15_H_18_O_9_	8.1	341.1030	341.1007	−6.9	CH	191.0513
3	Di-coumaroylquinic acid	C_25_H_23_O_12_	20.20	515.1077	515.1076	−3.2	A	353.0793, 191.0524
4	3-*O*-Galloylmalic acid	C_9_H_23_O_12_	21.17	285.0252	285.0282	12.3	A	241.9917
5	3-*O*-Caffeoylshikimic acid	C_9_H_23_O_12_	22.11	335.0713	335.0705	0.2	A	296.04122, 179.0316
6	5-*O*-Caffeoylshikimic acid	C_16_H_15_O_8_	22.27	335.0713	335.0719	0.6	A	296.04122, 179.0316
7	4-*O*-Galloylmalic acid	C_9_H_23_O_12_	22.42	285.0252	285.0284	7.8	A	240.9913
8	Rutin	C_12_H_25_O_11_	23.85	609.1361	609.1461	1.7	F	301.0632
9	3-*O*-Caffeoyl-5-*O*-malonylquinic acid	C_19_H_19_O_12_	23.91	439.0823	439.0836	3.0	A	341.1264
10	Cirsimaritin	C_17_H_14_O_6_	26.3	313.0658	313.0672	−1.1	F	271.0798, 627.14120 (2M-H)
11	Theaflavin-3-gallate	C_36_H_27_O_16_	26.0	715.1219	715.1210	1.2	A	339.04419
12	Unknown	C_30_H_27_O_15_	26.9	627.1508	627.1410	−15.2	-	271.0808
13	1-*O*,7-*O*-Digalloyl-D-sedoheptulose	C_21_H_21_O_15_	27.3	513.1038	513.0963	8.2	A	295.0549
14	Cretanin	C_20_H_22_O_13_	28.14	469.0927	469.0928	3.0	A	295.0435, 335.0622
15	Chrysoeriol	C_16_H_12_O_6_	29.75	299.0502	299.0520	4.2	F	271.0550
16	Galloyl citrate	C_13_H_11_O_11_	38.1	343.0400	343.0358	−12.4	A	299.0464
17	Na formiate (internal standard)	C_4_H_2_O_4_	38.6	112.9829	112.9856	3.1	A	-

CH = carbohydrates; A = aromatic; F = flavonoid.

**Table 5:** Total phenolic (TPC) and flavonoid content (FC) and antioxidant activity (FRAP; ORAC; DPPH) of *B. chilense, B. hastatum, B. magellanicum* and *B. penna-marina* extracts.

Assay	TPC (mg GAE/g)	FC (mg QE/g)	FRAP (µmol Trolox/g)	ORAC (µmol Trolox/g)	DPPH IC_50_ (µg/mL)
*B. chilense*	34.078 ± 0.010 *	52.959 ± 0.055	1589.752 ± 0.898 *	1567.615 ± 0.900 *	146.777 ± 0.022 *
*B. hastatum*	26.174 ± 0.080 *	29.929 ± 0.030 *	888.238 ± 0.647 *	1308.745 ± 0.865 *	205.143 ± 0.024 *
*B. magellannicum*	20.097 ± 0.050 *	52.408 ± 0.052	655.883 ± 0.620 *	1176.216 ± 0.805 *	260.965 ± 0.025 *
*B. penna-marina*	88.846 ± 0.020 *	128.662 ± 0.065 *	3301.847 ± 1.050 *	2677.519 ± 0.996 *	41.818 ± 0.005 *
Gallic acid ^#^	-	-	-	-	2.24 ± 0.04 *

In each trial, the values of three replicates are represented by their means ± SD. Statistically significant values are marked with * according to Tukey’s test (*p* ˂ 0.05). # Positive control.

**Table 6:** Enzyme inhibitory activity of B. chilense, B. hastatum, B. magellanicum and B. penna-marina extracts.

Assay	AChE IC_50_ (µg/mL)	BChE IC_50_ (µg/mL)
*B. chilense*	12.252 ± 0.028 *	ND
*B. hastatum*	8.311 ± 0.028 *	ND
*B. magellannicum*	10.713 ± 0.028 *	ND
*B. penna-marina*	9.572 ± 0.025 *	27.151 ± 0.078 *
Galantamine ^#^	0.266 ± 0.029 *	3.824 ± 0.024 *

In each trial, the values of three replicates are represented by their means ± SD. Statistically significant values are marked with * according to Tukey’s test (*p* ˂ 0.05). # Positive control. AChE is acetylcholinesterase; BChE is butyrylcholinesterase. ND, not detected.

## Data Availability

The data present in this study are available on request from the corresponding authors.
